# Editorial: Building resilience through healing communities

**DOI:** 10.3389/fpsyt.2024.1395869

**Published:** 2024-03-19

**Authors:** Jaswant Guzder, Geoffrey Walcott

**Affiliations:** ^1^ Department of Psychiatry, Division of Child Psychiatry and Division of Social and Cultural Psychiatry, McGill University, Montreal, QC, Canada; ^2^ Caribbean Institute of Mental Health and Substance Abuse, University of the West Indies, Kingston, Jamaica

**Keywords:** community resilience, indigenous interventions, task shifting and task sharing, mental health care, community based interventions

‘*Building resilience through healing communities’* the theme for this Research Topic focuses on global health initiatives implicated in reducing burden in LMIC populations (1). These pilot studies propose structural, relational, systemic and therapeutic alternatives relevant to under resourced LMIC (2). They are informed by interdisciplinary collaborations and ethnography, attuning ourselves to social suffering (3). Each paper promotes either community or individual well being through mental health tools or interventions grounded in task sharing or task shifting.

Innovations of task shifting and task sharing acknowledge that local solutions are central to strengthening community partnerships. Mental health research is enriched by these ethnographic explorations essential for understanding local health care planning factors and therapeutic needs. There is increasing interest in promoting local resilience through: understanding social determinants, the creative arts, equity of service access, and addressing challenges relevant to vulnerable populations (4).

Medical training often fails to address the importance of culturally adapted tools and approaches that appreciate the beliefs and realities of patients influencing wellbeing, resilience patterns and intervention. The Research Topic speaks to value of integrating social and culture in both individual and community mental health engagement. 

Transformative learning theory (TLT) (5), a model resonant with Paolo Friere’s theories (6) was applied by the Baheretibeb et al. as a useful approach to address and transform the dissonances between biomedical and traditional healing systems. Rural populations in Ethiopia with psychosis for instance, experience challenges accessing psychiatric care with only 58% accessing biomedical treatment. The treatment consequently falls on traditional healers of the Orthodox Christian Church who are experienced as less stigmatized than psychiatric care practitioners. Social and cultural discourses reflect on a task shifting process integrating complex locally relevant historical, relational, generational legacies, as well as spiritual traditions and diverse languages. Respectful feedback and exchange between the spiritual healers and research organizers led to productive collaborative partnerships. By exploring alternate ways of knowing and caring for seriously mentally ill patients. The Ethiopian team showed a reduction of to stigma and showed an increased referral to the psychiatric clinics.

Alemu et al. identified low rates of parental mental health literacy and stigma as a fundamental treatment barrier in a collaborative effort of Ethiopia, Kenya and Democratic Republic of Congo enriching our understanding of tools and evaluate ways to increase use of mental health services. Research is presented on the importance of parental health literacy as a contributing factor to promote support for vulnerable youth, especially those experiencing high mental health risk. This pilot successfully increased access to resources by the population of at risk youth by enhancing parental mental health literacy.

Promoting resilience in untreated LMIC child populations remains a priority (7) since children and youth mental health needs remain a relatively invisible unmet need (4).

The late Professor Frederick Hickling of University of West Indies introduced psycho-historiography (8, 9) as a group or individual psychotherapeutic approach which can be adapted across development from childhood and adulthood. This therapeutic modality evolved from a focus on decolonization processes and post slavery genocide issues embedded in Jamaica’s 400 years of slavery. De La Haye et al. with their team have been integrating this methodology with individual patients and piloted a trial of psilocybin assisted psychotherapy within the De La Haye Psilocybin Treatment Protocol (DPTP) (10). This protocol consists of eight weeks of psycho-historiographic therapy in outpatient sessions for treatment resistant trauma and depression patients.

Psychedelics are increasingly used in various Euro-North American psychiatric outpatient and inpatient settings (11). This is one of the first papers from an LMIC cohort using psilocybin in this unique application of psychotherapy processes. Hickling’s group process work culminated in a creative production of poetics and performance, while the psilocybin adaption is focussed on insight orientation with individual patients.

Creative arts modalities have continued to be transportable across cultural spaces and remains an accessible treatment modality for seriously mentally ill patients (12). These methods may use the facilitating modalities of visual arts, poetry, music, drama, dance and movement but also provide a space of protest and empowerment (13, 14).

The development of Zentangle as a simple mindfulness method with a cohort of treatment resistant or seriously mentally ill patients in recovery is explored by Stojcevski et al.. Further evaluation on Zentangle, is however warranted to assess the impact on the quality of life and skills for recovery. The method requires only brief training and can adapt to various age groups. Art methods can similarly provide mental health support as a task shifting strategy. The Bapu Trust project in Pune, India is a group who are a mental health NGO which has developed many projects integrating art based innovative task shifting for serious mental illness patients and community engagement.

While Qatar is a wealthy LMIC state their medical service development focussed mainly on tertiary care clinical services while access for community mental health resources remained undeveloped until 2016. Seventy five per cent of Qatar population are migrant workers predominantly from Nepal and Bangladesh, but this population remains underserviced in view of many structural and cultural challenges. Services remained less well developed currently for children, women and migrants.

Primary care also remains is underdeveloped with a shortage of trained mental health specialists and personnel. Women similarly remain underserved in mental health services though the study confirms significant community access improvements.

The research collaboration of Qatar with psychiatrists from Australia and Spain by Salinas-Perez et al., for example, was undertaken to progressively develop accessible community resources for serious mental illness. This development project has restructured service access for adults.

These papers though varied in their offerings, underline the possibilities of how each local community, region or cultural context provide solutions to the challenges of attending to the unmet LMIC mental health burden. Creative collaboration and ongoing refinement of best practices are therefore significant for mental health policy and its relation to human rights agendas.

## Author contributions

JG: Conceptualization, Writing – original draft. GW: Writing – review & editing.
